# Dandy-Walker malformation associated with hydrocephalus in a 15-month-old child: A case report with literature review

**DOI:** 10.1016/j.ijscr.2025.111229

**Published:** 2025-03-27

**Authors:** Turyalai Hakimi, Hasibullah Baha Nijrabi, Mohammad Yusuf Yadgari, Khalid Mohammad Qasem, Mohammad Anwar Jawed

**Affiliations:** a Department of Pediatric Surgery, Kabul University of Medical Science, Maiwand Teaching Hospital, Kabul, Afghanistan; b Department of Forensic Medicine, Kabul University of Medical Science, Kabul, Afghanistan; c Department of Orthopedic Surgery, Kabul University of Medical Science, Ali Abad Teaching Hospital, Kabul, Afghanistan

**Keywords:** Dandy walker, Hydrocephalus, Motor, Vermis, Posterior fossa, Head enlargement

## Abstract

**Introduction and importance:**

Dandy-Walker malformation is a posterior cranial fossa anomaly, characterized by the absence or hypoplasia of the vermis and cystic dilatation of the fourth ventricle, resulting in elevation of the tentorium and torcula. This condition can present with a broad range of neurological and developmental symptoms, highlighting the importance of early recognition and intervention to improve patient outcomes.

**Case presentation:**

This case describes a 15-month-old male presenting with head enlargement and motor dysfunction. A computerized tomography scan identified absence of the cerebellar vermis and ventriculomegaly. The patient underwent ventriculoperitoneal shunt placement, leading to significant improvement (head size, motor function) and complete recovery from convulsion over a six-month follow-up.

**Clinical discussion:**

The clinical presentation primarily originates from cerebellar dysfunction, impacting balance, coordination, vision, motor skills, cognition, and behavior. This condition occurs sporadically and is frequently associated with hydrocephalus. Treatment is centered on managing symptoms and related comorbidities.

**Conclusion:**

Dandy-Walker malformation is a congenital anomaly of the posterior fossa that causes a wide range of neurological and developmental challenges, primarily hydrocephalus. Effective management requires a multidisciplinary team, including pediatric surgeons and pediatricians, with timely consultation with a pediatric neurosurgeon and neurologist being essential.

## Highlights

• Dandy-Walker malformation, also known as Dandy-Walker syndrome, is a rare congenital neurological anomaly that affects cerebellar development.
• Clinical manifestations are nonspecific and depend on various factors, including the severity of hydrocephalus, intracranial hypertension, and related comorbidities.
• Treatment primarily aims to alleviate hydrocephalus and posterior fossa symptoms, often involving surgical interventions such as ventriculoperitoneal or cystoperitoneal shunting.

## 1 Introduction

Dandy-Walker malformation (DWM) is a rare neurological disorder characterized by agenesis or underdevelopment of the cerebellar vermis, leading to enlargement of the fourth ventricle. Its estimated incidence is 1 in 25,000 to 30,000 live births, with a higher prevalence in females [1]. Recent studies suggest that developmental defects in the roof of the rhombencephalon contribute to vermis hypoplasia and fourth ventricle dilation in DWM [2]. The clinical presentation varies and primarily depends on the severity of hydrocephalus. Treatment focuses on managing comorbidities, with shunt placement being the primary intervention to relieve hydrocephalus and fourth ventricle enlargement, thereby reducing intracranial pressure through cerebrospinal fluid diversion. This work has been reported in line with the SCARE 2023 criteria [3].

## 2 Case presentation

We present a 15-month-old male child from a northern province of the capital, referred to our pediatric surgery emergency room. The patient was born to a non-consanguineous couple, with all siblings reported as healthy.

According to the parents, around six months of age, the infant developed weak movements, convulsions, an enlarged head, lethargy, sunset eye sign, and excessive crying. Initially, the child was admitted to a public hospital in the capital, where conservative treatment was provided along with medical guidance. The same symptomatic treatment was administered twice at fixed intervals. The patient's reliance on repeated symptomatic treatment and limited access to specialized medical centers delayed definitive care until 15 months of age, ultimately resulting in hospital admission with pronounced symptoms.

**Fig. 1:**
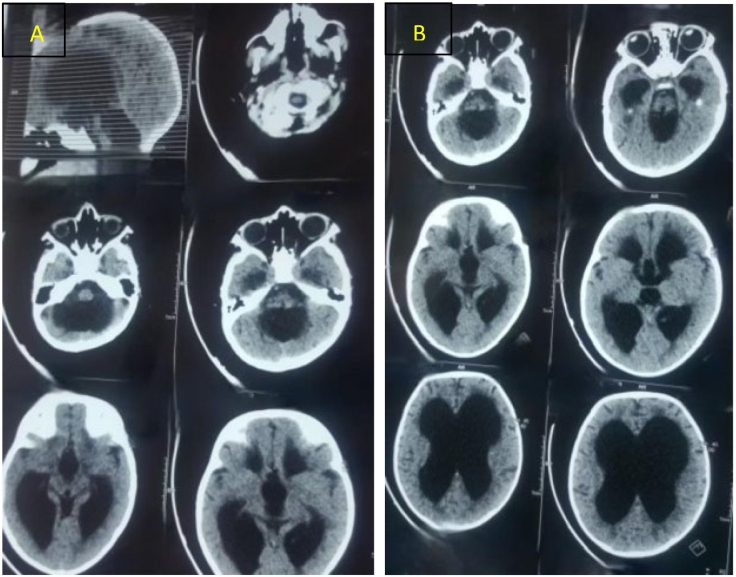
(A, B): Axial and sagittal brain CT scans demonstrating the absence of the cerebellar vermis and associated hydrocephalus.

During the physical examination, the patient was uncooperative, and Mongolian spots were noted in the lumbosacral region. As a result, a dermatology consultation was requested, leading to recommendations for appropriate management, including the use of sunblock and pigment-reducing therapy. Laboratory tests, biochemical investigations, and a brain computerized tomography scan (CT-Scan) were conducted. CT imaging revealed absence of the cerebellar vermis and ventriculomegaly, confirming the diagnosis of DWM associated with hydrocephalus (Fig. 1A, B).

The patient was prepared for definitive treatment, which included the placement of a ventriculoperitoneal (VP) shunt. Both the intraoperative and postoperative courses were uneventful, and the patient was discharged after a 48-hour postoperative hospital stay.

The patient underwent follow-up evaluations at the 3rd and 6th months to assess overall condition and clinical progress. A repeat brain CT scan revealed mild hydrocephalus due to brain atrophy. To evaluate intracranial pressure (ICP), fundoscopy was performed, which showed no papilledema, confirming normal ICP.

## 3 Discussion

DWM was first described by Dandy in 1914, with further details provided by Walker in 1942 [4]. While most cases of DWM occur sporadically, some may be associated with chromosomal aneuploidy, Mendelian genetic defects, or environmental factors such as congenital rubella and fetal alcohol exposure [5]. Additionally, DWM has been linked to various central nervous system (CNS) anomalies, including neural tube defects, holoprosencephaly, corpus callosum dysgenesis, and cortical developmental disorders [6].

The clinical presentation of DWM can be non-specific and influenced by factors such as the degree of hydrocephalus, intracranial hypertension, and associated comorbidities. Macrocephaly is the most common symptom during the first month of life. In syndromic forms of DWM, congenital anomalies involving the heart, face, limbs, gastrointestinal tract, or genitourinary system may also be present [7].

Prenatal diagnosis of DWM is possible by the 18th week of gestation using ultrasonography (USG), as the cerebellar vermis is fully developed by this stage. Magnetic resonance imaging (MRI) can confirm the diagnosis and, along with karyotype analysis, helps differentiate DWM from other CNS disorders. In cases with an enlarged cisterna magna, measurements of the brainstem-tentorium (BT) and brainstem-vermian (BV) angles are useful diagnostic markers. A BV angle between 18 and 30 suggests Blake's pouch defect, while a BV angle below 18 indicates a more severe anomaly. A BT angle of 12–44 is considered normal, whereas a BT angle of 45 or greater supports a diagnosis of DWM [8,9].

Surgical intervention is the preferred treatment for DWM to alleviate clinical manifestations and comorbidities associated with posterior fossa cysts and hydrocephalus. Treatment options include minimally invasive endoscopic third ventriculostomy (ETV) and the placement of a ventriculoperitoneal (VP) or cystoperitoneal (CP) shunt [6,10]. Without treatment, 50 % of children with hydrocephalus associated with DWM die before the age of three, and only 20 % to 23 % survive into adulthood. Visual, auditory, and motor deficits are common in adults with untreated hydrocephalus [11]. ETV, a feasible procedure with fewer complications, is not accessible in most of our public hospitals.

In the index case, an early postnatal diagnosis was made, and conservative management was initially pursued with regular medical guidance at a public hospital. The decision to proceed with surgical intervention aimed to reduce ICP and manage symptoms. However, continuous follow-up is essential to monitor for potential symptom recurrence, particularly due to shunt malfunction, ensuring timely intervention when necessary. In our case, follow-up brain CT scans after VP shunt placement revealed mild brain atrophy, possibly due to pressure on the brain before the procedure.

In many underdeveloped countries, where a significant proportion of women lack formal education or are illiterate, it is crucial for physicians to provide thorough guidance on post-surgical care, particularly in managing the child's shunt. Additionally, genetic counseling for parents of children with DWM is an important consideration in these regions. In Afghanistan, where consanguineous marriages are common and premarital screenings for hereditary or debilitating conditions are often lacking, genetic assessments play a vital role in family planning. Addressing all of these will take a considerable amount of time in this country. Furthermore, regular monitoring of the child's nutritional status and growth is a key component of long-term follow-up.

## 4 Conclusion

This case highlights the significance of timely diagnosis of DWM in preventing its adverse effects on developing brain and related neurological complications. Early intervention is essential to reducing hydrocephalic pressure on the brain parenchyma. Furthermore, genetic counseling plays a critical role, especially for families with multiple children, in minimizing the risk of recurrence.

## Author contribution

Turyalai Hakimi (TH) conceptualized the manuscript, reviewed the literature, and wrote the original draft. Hasibullah Baha Nijrabi (HBN), Mohammad Yusuf Yadgari (MYY), and Khalid Mohammad Qasem (KMQ) designed the study and edited the manuscript. TH and Mohammad Anwar Jawed (MAJ) performed the surgical procedure (ventriculoperitoneal [VP] shunt application). TH also edited the manuscript and supervised the entire study process. All authors read and approved the final manuscript.

## Consent

Written informed consent was obtained from the patient's parents for publication of this case report and accompanying images. A copy of the written consent is available for review by the Editor-in-Chief of this journal on request.

## Ethical approval

Not applicable, this type of work is considered as research paper, but doesn't need ethical approval by our institution review board (The institution review board of the Kabul University of Medical Science).

## Guarantor

Turyalai Hakimi.

## Research registration number

Not applicable.

## Funding

No funds or grants.

## Conflict of interest statement

The authors declare that they have no known competing financial interests or personal relationships that could have appeared to influence the work reported in this paper.
